# Photodynamic Diagnosis For Superficial Bladder Cancer: do All Risk-Groups Profit Equally From Oncological and Economic Long-Term Results?

**DOI:** 10.4137/cmo.s1012

**Published:** 2009-04-30

**Authors:** Wolfgang Otto, Maximilian Burger, Hans-Martin Fritsche, Andreas Blana, Wolfgang Roessler, Ruth Knuechel, Wolf F. Wieland, Stefan Denzinger

**Affiliations:** 1 Department of Urology, University of Regensburg, Germany.; 2 Institute of Pathology, RWTH Aachen, Germany.

**Keywords:** superficial bladder cancer, urothelial carcinoma, photodynamic, transurethral resection, recurrence rate, cost analysis

## Abstract

**Objective:**

Photodynamic diagnosis (PDD) of superficial bladder cancer decreases recurrence rates. We present oncological results of a randomized, prospective study, comparing transurethral resection (TUR) performed under conventional white light (WL) with PDD. The follow-up period is the longest reported to date. As costs might be reimbursed by prolonged recurrence-free survival in certain patients cost analysis in regard to risk-groups was performed.

**Material and methods:**

Using chi-square test and log-rank test we compared recurrence rates of 103 patients after WL-TUR and of 88 patients after PDD-TUR. Cost analysis was performed according to risk-groups of recurrence.

**Results:**

Mean follow-up was 99 months. Recurrence rate was 57% in WL vs. 28% in PDD (p < 0.001). Costs incurred by subsequent TUR averaged € 2310 per WL patient vs. € 713 per PDD patient. Savings per patient by PDD amounted to € 1597. PDD costs were reimbursed in low, intermediate and high risk patients, respectively.

**Conclusions:**

PDD-TUR is significantly superior to conventional WL-TUR in terms of recurrence rate. While economic benefit is most prominent in intermediate risk patients, PDD related costs are reimbursed in all risk-groups.

## Introduction

About 70% of transitional cell carcinomas of the bladder are superficial.^1^ Despite macroscopically complete tumor removal including muscular tissue, over two-thirds of patients undergoing transurethral resection (TUR) with white light experience recurrence. In approximately 15% of cases, stage progression to muscle-invasive tumors is observed requiring radical surgery.^2^ The high recurrence rate has been partly attributed to the poor macroscopical visibility of scattered tumor tissue, e.g. carcinoma in situ (CIS), that might be overlooked in conventional TUR leading to recurrence.

Urological research has long sought a practicable technique improving recognition of malignant urothelial tumors. In 1992, one such clinically applicable procedure was introduced, intravesical application of 5-aminolevulinic acid (5-ALA) with subsequent photodynamic diagnostics (PDD).^3^ PDD has been reported to facilitate detection of flat, small papillary and remnant lesions poorly visible under conventional white light.^4^,^5^ 5-ALA instilled into the bladder prior to TUR is metabolized to protoporphyrin-IX in the urothelial cell. Whilst in normal cells, the enzyme ferrochelatase transforms this fluorescent agent into heme, this step is impaired in tumor cells, inflamed or granulating tissue: Protoporphyrin-IX accumulates producing reddish fluorescence under blue light (345–440 nm).^6^

The first publications on PDD mainly focused on its diagnostic value with sensitivities over 97% and specifities ranging between 35% and 70%.^5^,^7^–^12^ The high sensitivity lead some authors to call for general application of PDD in the diagnosis of bladder cancer.^13^ Critics however rejected PDD due to its low specifity.

Randomized, prospective clinical trials^14^,^15^ however reported a statistically significant reduction of the residual tumor rate using PDD. Our institution performed a randomized prospective study in order to clarify whether recurrence rate could be reduced by PDD.

Superficial bladder cancer imposes enormous costs on public health systems due to its life-long character. By reducing the frequency of recurrences PDD has been suggested to ease this economic burden.^16^,^17^ However as PDD is related to specific expenditures its use may not be justified on a larger scale but in those patients with obvious benefit from PDD only. Multifocality, previous bladder cancer, tumor stage and histologic grade have been recognized as predictors of recurrences and have been reported to stratify risk-groups.^18^

In the present study we report the longest experience with recurrences following WL versus PDD. Hospitals are compensated for surgical procedures according to the DRG (diagnosis related groups) system providing equal and transparent funding. We analyzed all costs related to subsequent TURs and the use of PDD. Of focal interest was the question if reimbursement of PDD-related expenditures can be achieved in all risk-groups and so every patient with superficial bladder cancer could profit from the use of PDD.

## Material and Methods

Ethical approval and written informed consent from each patient were obtained. From May 1997 until August 2000, 301 patients presenting with lesions suspicious for bladder cancer in urethro-cystoscopy were randomized into TUR with conventional white light cystoscopy (n = 150) or TUR supplemented by PDD with 5-ALA (n = 151) as previously reported.^19^

Six weeks after the initial TUR all patients underwent repeat WL resection to evaluate residual tumor rate. All specimen were graded according to the 1994 WHO classification and staged according to the 1997 TNM system.^20^,^21^ Patients with muscle-invasive urothelial carcinoma (≥T2) or benign histological findings were excluded from the study. Intravesical recurrence prophylaxis was performed according to the guidelines of the American Urological Association (AUA) for treatment of superficial bladder cancers. Whilst solitary primary tumors pTaG1–G2 did not receive recurrence prophylaxis, patients with multifocal bladder lesions staged pTaG1–G3 and pT1G1–G2 received mitomycin (MMC) treatment. Primary pT1G3 tumors, carcinoma in situ plus patients who did not respond to MMC received instillation therapy with 6 weekly courses of bacillus Calmette-Guérin (BCG).^22^ In the case of recurring pT1G3 and Cis, patients were advised to undergo radical cystectomy. The patients attended follow-up examinations involving white light cystoscopy and cytology at quarterly intervals, the first follow-up being 3 months after secondary transurethral resection. Recurrence was verified by TUR and histopathology.

Recurrence rates were evaluated for each risk-group as defined by respective guidelines issued by the European Association of Urology (EAU).^18^ Expenditures related to subsequent TURs were calculated based on the current German DRGs. Costs related to PDD were evaluated. Chi-square and log-rank tests were used to determine statistical significance between the groups, values <5% were considered significant.

## Results

### Oncological long-term outcome

Data from 103 of the 150 patients in the white light arm (WL) could be evaluated, 47 were excluded lacking malignancy (n = 21), presenting with muscle-invasive urothelial carcinoma (n = 24) or refusing post-operative follow-up (n = 2). Of the 151 patients randomized into the fluorescent diagnostic study arm (PDD), 88 could be evaluated after excluding 63 patients (38 + 23 + 2).

Mean age was 70 years (range: 32 to 79 years) and 68 years (range: 31 to 88 years) in WL and PDD, respectively. Distribution of gender, tumor stage, grade and prognostic groups according to the EAU was similar between the two patient groups. However distribution of multifocality was disadvantageous for PDD (Table 1). Residual tumor rate was 25.2% in WL versus 4.5% in PDD (p < 0.001). Reresection did not show statistically significant differences in upgrading between both groups. Also percentage of prophylaxis—with MMC in 30% (WL) and 36% (PDD), respectively, and with BCG in 29% (WL) and 36% (PDD), respectively—did not show statistically significant differences between the risk-groups in both study arms (p = 0.81).

**Figure 1.**
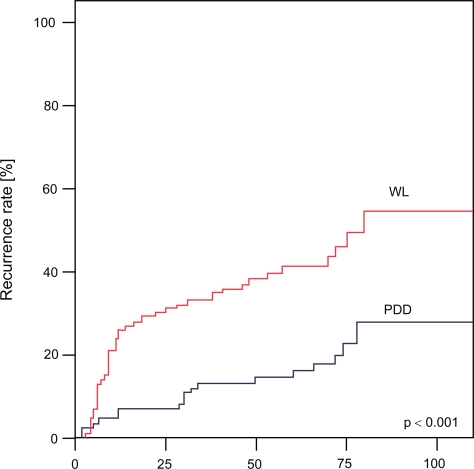
Analysis of recurrence rates after conventional white light (WL) transurethral resection and photodynamic diagnostics (PDD) in study patients.

Mean follow-up was 102 months (76–110 months) in WL and 98 months (73–108 months) in PDD, respectively. Throughout the follow-up period 57% recurrences were observed in WL versus 28% in PDD (Fig. 1; log rank test: p < 0.001). The recurrence rate after 25, 50, 75 and 100 months in WL was 32%, 39%, 48% and 57%, respectively, compared to 9%, 17%, 22% and 28% in PDD. Superiority of PDD was confirmed for all risk-groups in sub-group analysis (Table 2). 4 patients in WL (3.9%) and 3 patients in PDD group (3.4%) developed muscle-invasive progression with need for cystectomy.

### Economic long-term results

During follow-up, 238 further TURs were performed due to suspicious findings in WL (2.3 per patient), as opposed to 104 TURs (1.2 per patient) in PDD. Deducting TURs revealing false positive lesions, 1.32 and 0.33 TURs per patient were performed for malign findings in WL and PDD, respectively.

5-ALA was marketed at € 95 per instillation and material and time required for single-use catheterization summed up to € 30. Considering a depreciation over 10 years and 50 uses per year, € 10 were determined as equipment related costs per use concerning equipment costs at about € 5000. Thus PDD-related costs amounted to € 135 per patient. In the current German DRG system TUR burdens the public health care system with € 1750. Considering resections with true positive findings only TUR related expenditures were € 2310 (1.32 × DRG) in WL versus € 713 (0.33 × DRG + € 135) in PDD after 100 months, respectively. Thus about 192 € (€ 1597:100 × 12) were saved per PDD patient per year followed-up in the present series.

**Figure 2.**
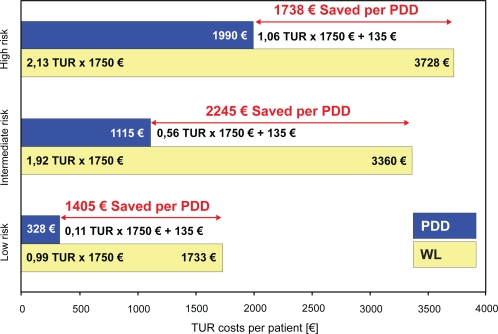
Calculation of white light (WL) transurethral resection (TUR) and photodynamic diagnostics (PDD) related costs in low, intermediate and high risk patients.

In the subgroup analysis an economic advantage of PDD was noted in all risk-groups (Fig. 2). However it was most pronounced in intermediate risk patients, as € 1405, € 2245 and € 1738 were saved by the use of PDD in low, intermediate and high risk patients, respectively.

## Discussion

Overlooking of smaller flat and papillary lesions in conventional white light during primary transurethral resection poses a major problem in the treatment of bladder cancer. Various studies report residual tumor rates after TUR of superficial bladder tumors between 30% and 70%.^2^,^12^,^23^–^25^ Thus repeat resection has been advocated in some cases approximately 6 weeks after primary TUR for monitoring and completion of tumor resection.^26^ The outcome of repeat resection can serve as a parameter for the quality of the 5-aminolevulinic acid (5-ALA)-induced fluorescence diagnostics. The comparison of residual tumor rates after WL and PDD was the first test parameter in the presented controlled, randomized clinical trial. In the WL arm residual tumor rate was 25.2% versus a mere 4.5% in PDD (p < 0.001).^19^ This finding is in accordance with previous studies by Riedl et al. and Kriegmair et al.^14^,^27^ The rate of upgradings in reresection did not differ statistically significant between both groups.

However in contrast to a reduction in residual tumor rate only reduction in recurrence rate poses a clinically relevant advantage. A first interims analysis of this study after 42 months showed PDD to reduce recurrence rates.^5^ Daniltchenko et al. described recurrence rates after 60 months of 59% in PDD and 75% in WL, respectively.^16^ However, only long-term data can evaluate the true benefit of PDD. Thus we present the longest follow-up period reported today. After a follow-up of 100 months patients in the PDD arm showed a recurrence rate of 28% as opposed to 57% in the WL arm (p < 0.001). This finding was consistent in all risk-groups.

While the oncological outcome seems to benefit from PDD the reduction in tumor recurrences might also reduce the economic burden imposed on public health systems by subsequent surgery. In a long-term analysis, costs per patient were reduced by about € 1597. The additional costs of € 135 related to PDD are reimbursed by far. While previous studies with shorter follow-up periods have not taken the influence of risk factors into account, the present contribution stratified economic analysis according to risk factors as defined by the EAU.^9^,^17^,^18^ We were able to show that PDD holds an economic benefit and PDD related costs are reimbursed even in low risk cases.

Recently 5-ALA ester hexaminolevulinic acid (Hexvix, GE-Healthcare, Munich, Germany) was licensed in Europe. It has been suggested to promote the results of 5-ALA as the standard in PDD.^28^

In conclusion photodynamic diagnostics significantly reduces recurrence rates in the longest follow-up period reported to date. This effect was apparent independently of the patients' prognostic risk-group. PDD seems to hold oncological and economical benefits. In the present series subsequent costs were reduced by almost € 1600 per patient by the use of PDD and reimbursement of PDD-related costs was achieved even in low risk patients by far.

## Figures and Tables

**Table 1.** Characteristics of study patients.

	**WL arm (n = 103)**	**PDD arm (n = 88)**	**p value**
**First occurrence of BC [%]**	81.6	69.3	p = 0.06
**Staging [%]**			p = 0.75
pTaG1	41.7	42.0	
pTaG2	28.2	30.7	
pTaG3	1.0	2.3	
pT1G1	0	1.1	
pT1G2	12.6	6.8	
pT1G3	11.7	11.4	
pTis	4.9	5.7	
**Focality [%]**			p = 0.03
solitary tumor	76.7	62.5	
multifocal tumors	23.3	37.5	
**Risk groups [%]**			p = 0.39
low (solitary/first occurred pTaG1/2, pT1G1)	48.5	35.2	
intermediate (all other stages)	34.0	45.5	
high (pTaG3, pT1G3, pTis)	17.5	19.3	

**Abbreviations:** BC, bladder cancer; PDD, photodynamic diagnosis; WL, white light cystoscopy.

**Table 2.** Recurrence rates of study patients after follow-up periods of 25, 50, 75 and 100 months stratified in low, intermediate and high risk patients.

	**WL arm [%]**	**PDD arm [%]**	**p value (log-rank test)**
**Study patients** (WL n = 103, PDD n = 88)			p < 0.001
recurrence rate after 25 months	32	9	
recurrence rate after 50 months	39	17	
recurrence rate after 75 months	48	22	
recurrence rate after 100 months	57	28	
**High risk group** (WL n = 18, PDD n = 17)			p = 0.02
recurrence rate after 25 months	48	18	
recurrence rate after 50 months	61	31	
recurrence rate after 75 months	79	39	
recurrence rate after 100 months	85	56	
**Inter risk group** (WL n = 35, PDD n = 40)			p = 0.02
recurrence rate after 25 months	40	14	
recurrence rate after 50 months	49	20	
recurrence rate after 75 months	58	30	
recurrence rate after 100 months	60	33	
**Low risk group** (WL n = 50, PDD n = 31)			p = 0.003
recurrence rate after 25 months	16	2	
recurrence rate after 50 months	28	5	
recurrence rate after 75 months	45	13	
recurrence rate after 100 months	48	19	

**Abbreviations:** PDD, photodynamic diagnosis; WL, white light cystoscopy.
